# Hepatic Dysfunction as a Paraneoplastic Manifestation of Metastatic Prostate Adenocarcinoma

**DOI:** 10.1177/2324709614539927

**Published:** 2014-06-24

**Authors:** David Kato, Chinemerem Okwara, Christopher Moreland, Allan Parker

**Affiliations:** 1 University of Texas Health Science Center, San Antonio, TX, USA

**Keywords:** Stauffer’s syndrome, metastatic prostate adenocarcinoma, androgen deprivation therapy

## Abstract

Cholestasis is a general feature of intrahepatic or extrahepatic biliary obstruction by various mechanisms including cirrhosis, stricture, choledocholithiasis, hepatitis, and neoplasms. Neoplasms can directly impinge on the hepatobiliary tree resulting in bile stasis. Stauffer’s syndrome is another variant of this neoplastic process that can cause cholestasis and liver enzyme elevation without any direct hepatobiliary obstruction, and is thus categorized as a paraneoplastic syndrome of unclear pathophysiology. We report a first case of metastatic prostate adenocarcinoma with features of Stauffer’s syndrome that reversed completely on androgen deprivation therapy. This is in contrast to a previously reported case of Stauffer’s syndrome due to metastatic prostate adenocarcinoma, which reversed partially to androgen deprivation therapy. Our case demonstrates the importance of early recognition of Stauffer’s syndrome and underlying neoplasms in patients who present with cholestasis without clear evidence of intrahepatic or extrahepatic biliary obstruction, which may lead to early initiation of treatment.

## Case Report

A 60-year-old African American man presented to our hospital with a 6-month history of progressively worsening fatigue, 30-pound weight loss, jaundice, pruritis, dark urine, and straining on urination. He reported no previous intravenous drug use. His physical exam revealed nontender lymphadenopathy involving the anterior cervical, axillary, and inguinal areas. The rectal exam revealed irregularity of the left prostatic border. Initial laboratory results were significant for hyperbilirubinemia (9.3 mg/dL) with direct bilirubin predominance (7.9 mg/dL), as well as elevated aspartate aminotransferase (200 IU/L), alanine aminotransferase (169 IU/L), and alkaline phosphatase (1803 IU/L) with predominant hepatic origin evidenced by elevated liver isoenzyme (1352 IU/L) and γ-glutamyltransferase (2152 IU/L). Hepatitis, HIV, autoimmune, and acetaminophen toxicity evaluations were negative. Prostate-specific antigen was elevated (41.4 ng/mL).

Computed tomography of the chest, abdomen, and pelvis showed markedly enlarged prostate glands with metastases involving the bones, lungs, and lymph nodes but sparing the hepato-cholangio-pancreatic organs. Magnetic resonance cholangiopancreatography revealed normal liver morphology with multiple hypointense T1/hyperintense T2-weighted signal intensity lesions scattered throughout the liver, thought to represent hepatic cysts, with no intrahepatic or extrahepatic biliary duct dilatation to suggest obstruction.

**Figure 1.**
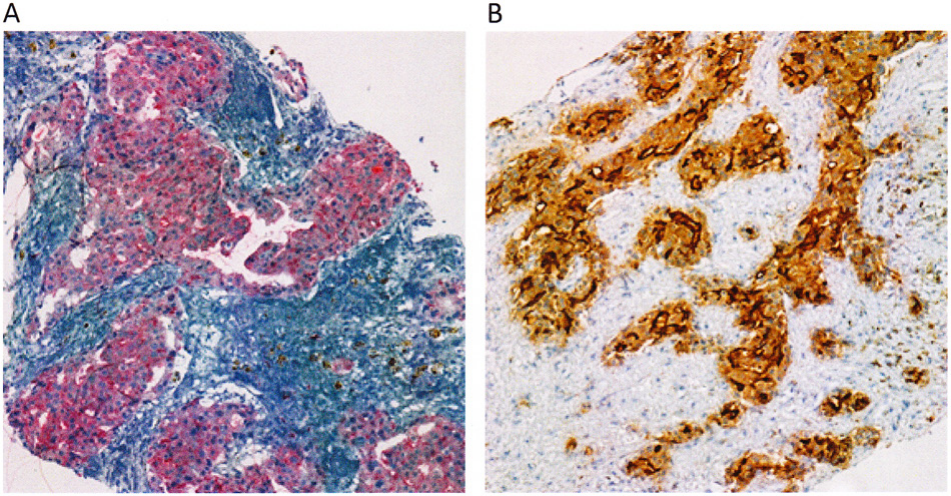
Immunostain of left external iliac lymph nodes: (A) Positive prostate-specific antigen stain; (B) Positive CD-10 stain.

The patient underwent fine needle aspiration of the left external iliac lymph nodes; pathology revealed malignant cells consistent with metastatic adenocarcinoma of prostatic origin. Immunostaining for prostate-specific antigen (Figure 1A) and CD-10 (Figure 1B) were positive.

**Figure 2.**
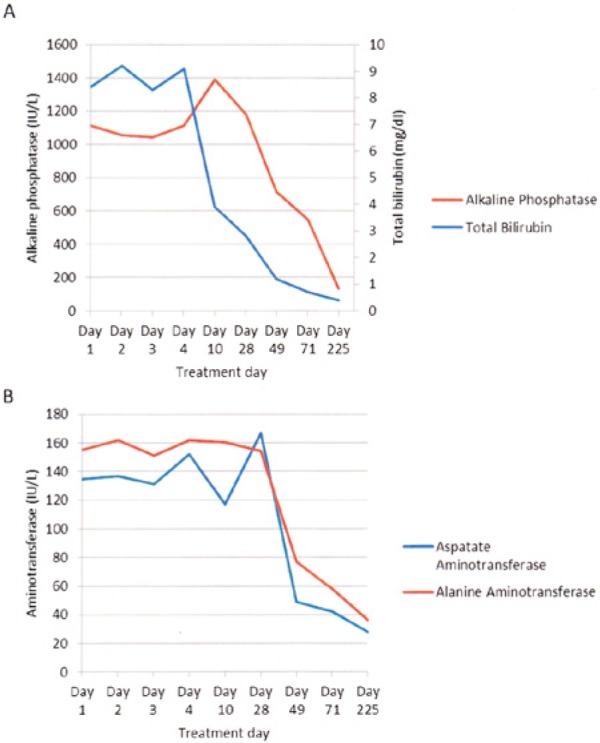
Liver function response to androgen deprivation therapy. Bicalutamide was given daily except for between day 4 and day 28. Leuprolide was given on day 49 and day 71. (A) Rapid decline in total bilirubin and alkaline phosphatase after treatment day 4 and day 28, respectively. (B) Rapid decline in aspartate aminotransferase and alanine aminotransferase after treatment day 28.

Following oncology consultation, the patient began androgen deprivation therapy for inoperable metastatic prostate cancer. He took bicalutamide, an androgen receptor blocker, for 4 days during the hospitalization but stopped for the next 23 days since he failed to pick up the medications. Bicalutamide was restarted on treatment day 28. Additionally, he received leuprolide, a GnRH analog, on treatment day 49 and day 71. The patient responded well to the treatment as the liver function tests normalized over time (Figure 2). Improvement in laboratory values paralleled clinical improvement, as the lymph nodes became nonpalpable and jaundice and pruritus completely resolved.

## Discussion

Stauffer’s syndrome, a paraneoplastic syndrome resulting in liver enzyme derangement, was initially introduced by Dr Maurice H. Stauffer, who witnessed a reversal of hepatic dysfunction following a resection of renal cell carcinoma.^1^ Since its original discovery, more literature has emerged supporting Stauffer’s syndrome in various types of neoplasms. Thus far, there are only 6 reported cases of adenocarcinoma as a source of Stauffer’s syndrome.^2-7^ Only one case of metastatic prostate adenocarcinoma demonstrated partial resolution of hepatic dysfunction with androgen deprivation therapy.^2^ Our case is the first report, to our knowledge, of metastatic prostate adenocarcinoma with CD-10-positive stain that responded to androgen deprivation therapy, resulting in a complete reversal of hepatic dysfunction in all measured categories. Of note, the total bilirubin level reversed early in his course, despite interruption of androgen deprivation therapy, which may be explained by bicalutamide’s long half-life of 6 days. The pathophysiology of Stauffer’s syndrome remains largely unexplained, but interleukin-6 is thought to play a crucial role.^8^ Overall, Stauffer’s syndrome is a rare complication of many types of cancer; however, liver dysfunction without obvious cause in the presence of concomitant neoplasm must be readily recognized as a possible paraneoplastic process.
